# Host, Pathogen, and Pulmonary Anatomy in Pneumonia: An Integrated Imaging and Clinical Perspective

**DOI:** 10.3390/diagnostics16111605

**Published:** 2026-05-25

**Authors:** Teodora Biciusca, Thomas J. Vogl, Liv Goedeking, Lisa Joy Juergens, Elena Höhne, Anna-Sophia Dietrich, Andreea-Ioana Nica, Jennifer Gotta, Aynur Gökduman, Simon S. Martin, Mara Balteanu, Mirela-Elena Popescu, Sorina-Ionelia Stan, Viorel Biciușcă

**Affiliations:** 1 Department of Radiology, University Hospital, Goethe University Frankfurt, 60596 Frankfurt am Main, Germany; 2 Department of Pneumology, Faculty of Medicine, Titu Maiorescu University, 031593 Bucharest, Romania; 3 Doctoral School, University of Medicine and Pharmacy of Craiova, 200349 Craiova, Romania; 4 Department of Internal Medicine, Faculty of Medicine, University of Medicine and Pharmacy of Craiova, 200349 Craiova, Romania

**Keywords:** pneumonia, pathogens, host factors, imaging, pulmonary distribution, empirical therapy

## Abstract

Pneumonia remains a major global health concern, particularly among elderly and immunocompromised populations, and is associated with substantial morbidity, mortality, and healthcare burden. It is a heterogeneous infectious disease caused by a wide variety of microorganisms, resulting in diverse imaging manifestations and characteristic patterns of pulmonary distribution. Despite advances in microbiological diagnostics and imaging, the literature still lacks a comprehensive overview integrating the imaging characteristics of pneumonia with pathogen-specific features and host susceptibility. A structured literature search of PubMed, Scopus, and Web of Science was conducted for studies published between 2000 and 2026 focusing on key pathogen groups and their radiological patterns. The reviewed evidence indicates that pulmonary distribution is determined by a complex interplay between infection routes, lung anatomy and physiology, host defense mechanisms, patient-specific, and environmental exposures. Radiological patterns, including lobar, bronchopneumonic, interstitial, necrotizing, abscess-forming, and cavitating forms, may correlate with pathogen type, disease severity, and host vulnerability. This integrative approach emphasizes the importance of correlating imaging findings with clinical presentation and patient risk factors to support early etiological assessment and guide empirical therapy. Improved understanding of the determinants of pulmonary distribution may facilitate personalized management, rapid clinical decision-making in emergencies and hospital settings, and improved clinical outcomes.

## 1. Introduction

Pneumonia is an infectious disease of the lung caused by a broad spectrum of pathogens, including bacteria, viruses, fungi, and atypical organisms, with bacterial pneumonia remaining the most clinically relevant form in both community- and hospital-acquired settings. The disease demonstrates considerable heterogeneity in clinical presentation, radiological manifestations, and severity, largely influenced by pathogen-related characteristics and host factors. Despite advances in antimicrobial therapy, microbiological diagnostics, vaccination strategies, and thoracic imaging, pneumonia remains a major cause of morbidity and mortality worldwide, particularly among elderly individuals and patients with chronic comorbidities or immunosuppressive conditions [1].

In the European Union, pneumonia is responsible for approximately 230,000 deaths annually and remains one of the leading causes of infectious mortality [2]. Mortality rates vary substantially among countries due to differences in healthcare access, vaccination coverage, prevalence of chronic diseases, and public health policies [3]. Community-acquired pneumonia (CAP) continues to impose a considerable healthcare burden, particularly through hospitalization, long-term morbidity, and increased mortality among older adults [4,5,6].

The risk of CAP significantly increases with advancing age and with chronic comorbidities such as chronic obstructive pulmonary disease (COPD), asthma, diabetes mellitus (DM), congestive heart failure (CHF), cancer, immunosuppression, and smoking [3]. As the global population continues to age and the prevalence of complex comorbidities increases, pneumonia is increasingly encountered in clinically vulnerable patient populations with heterogeneous imaging manifestations and overlapping etiologies [7,8,9].

Thoracic imaging plays a central role in the diagnosis, severity assessment, and therapeutic monitoring of pneumonia. However, radiological findings are frequently non-specific and may overlap substantially among infectious etiologies. Accurate interpretation therefore requires integration of imaging findings with pathogen-related characteristics, host immune status, epidemiological exposure, and clinical presentation.

Although numerous studies have separately evaluated pathogen-specific manifestations, host susceptibility, or radiological findings, the literature still lacks a comprehensive integrative overview correlating these factors within a unified clinicoradiological framework. This narrative review aims to address this gap by synthesizing current evidence regarding the determinants of pulmonary distribution, imaging manifestations, and pathogen-specific features of pneumonia, with emphasis on the interaction between microbial, anatomical, and host-related factors. Accordingly, this work is organized into three main parts: (1) factors determining the distribution of pneumonia, (2) imaging manifestations of pneumonia, and (3) pathogen-specific clinicoradiological features.

## 2. Materials and Methods

This manuscript was designed as a narrative review using a structured literature search methodology. Relevant studies were identified through searches of PubMed, Scopus, and Web of Science databases covering publications from January 2000 to March 2026 in English and German. This time frame was selected to capture contemporary evidence reflecting advances in thoracic imaging, microbiological diagnostics, and the evolving epidemiology of pneumonia over the past two decades.

The search strategy combined controlled vocabulary and free-text terms related to pneumonia etiology, host factors, and imaging manifestations using Boolean operators. The following search terms were used:

- (“pneumonia” OR “community-acquired pneumonia” OR “nosocomial pneumonia”);

AND

- (“bacterial etiology” OR “atypical pneumonia” OR “opportunistic infection”);

AND

- (“risk factors” OR “comorbidity” OR “immunocompromised”);

AND

- (“imaging” OR “chest radiograph” OR “computed tomography” OR “lobar distribution”).

The search strategy was adapted for each database to optimize sensitivity and reproducibility, including the use of database-specific controlled vocabulary (e.g., Medical Subject Headings [MeSH] in PubMed), free-text keywords, and appropriate Boolean operators. Study selection followed a two-step process: first, titles and abstracts were screened for relevance; second, full-text articles were assessed for eligibility based on predefined criteria.

The inclusion criteria were as follows:

1. Original studies, systematic reviews, narrative reviews, or clinical guidelines on community-acquired or hospital-acquired pneumonia;
2. Studies evaluating relationships between pathogens, host factors, and pulmonary distribution patterns;
3. Articles reporting imaging findings (radiography and/or computed tomography) in pneumonia;
4. Studies involving adult or pediatric populations, including immunocompromised patients and individuals with chronic lung diseases.

The exclusion criteria were as follows:

1. Animal or in vitro studies without direct clinical relevance;
2. Studies lacking sufficient clinical, microbiological, or imaging data;
3. Conference abstracts without full-text availability and unpublished data;
4. Non-English and non-German publications.

Study selection was independently performed by two reviewers to improve consistency and reduce selection bias. Disagreements were resolved by consensus. No formal risk-of-bias assessment tool was applied due to the narrative design of the review and the heterogeneity of the included study types.

## 3. Results

### 3.1. Factors Determining the Topographic Distribution of Pneumonia

#### 3.1.1. Main Routes of Pulmonary Infection

The lungs can be infected via several distinct routes, and the entry pathway of a pathogen significantly influences the topographic distribution of pneumonia. The main infection routes are well established in the literature; however, more recent studies have further refined the understanding of their relationship with specific radiological distribution patterns, particularly in vulnerable patient populations [10].

The respiratory route remains the most common mechanism of infection, involving inhalation of contaminated droplets or aerosols from infected individuals. This leads to colonization of the airways and alveoli and is typically associated with pathogens such as *Streptococcus pneumoniae*, *Mycoplasma pneumoniae*, and a wide range of respiratory viruses [11,12].

Aspiration represents another major route, particularly in patients with impaired consciousness, dysphagia, or reduced airway protection reflexes [13]. Recent evidence has further emphasized its strong association with dependent lung involvement and its increased prevalence in elderly and neurologically impaired patients. Aspiration-related pneumonia most frequently affects dependent lung regions, with a predilection for the posterior segments of the lower lobes. In intubated or mechanically ventilated patients, altered airway clearance and microaspiration contribute to ventilator-associated pneumonia, which commonly involves similar dependent regions [14].

Hematogenous spread is less frequent and occurs secondary to systemic infections such as endocarditis or osteomyelitis. In these cases, pulmonary involvement is typically multifocal and bilateral, without a consistent lobar distribution [15,16].

Rare mechanisms include direct extension from adjacent infectious sites, such as hepatic, mediastinal, or thoracic abscesses, and traumatic inoculation following penetrating chest injuries [17].

#### 3.1.2. Lung Anatomy and Physiology Relevant to Pulmonary Infection

A structured understanding of lung anatomy and physiology is essential for interpreting the distribution patterns of pneumonia. The bronchial tree branches into main, lobar, segmental, and subsegmental bronchi, which determine airflow distribution and influence the regional deposition of inhaled pathogens [18]. Anatomical asymmetry between the main bronchi contributes to infection localization. The main right bronchus is shorter and more vertical than the left, facilitating preferential entry of aspirated material into the right lung, particularly the lower lobe [19].

Regional differences in ventilation and perfusion further contribute to disease localization. Upper lobe apices are relatively well ventilated but less perfused, creating conditions that may favor aerobic organisms such as *Mycobacterium tuberculosis*, whereas dependent basal regions are more prone to secretion retention and infection with pathogens such as *Haemophilus influenzae* due to reduced ventilation efficiency [20,21].

Patient positioning significantly modifies aspiration patterns. In the supine position, aspirated material tends to reach posterior segments of the right upper lobe and the apical segment of the right lower lobe. Upright positioning favors basal involvement, whereas lateral decubitus positioning results in dependent-sided infiltration patterns [22,23].

In mechanically ventilated patients, airflow dynamics and artificial airways alter normal clearance mechanisms, contributing to ventilator-associated pneumonia (VAP), which typically develops after prolonged ventilation in intensive care settings [24].

#### 3.1.3. Host Defense Mechanisms

Recent studies have highlighted the role of pulmonary defense mechanisms in shaping both susceptibility to infection and the distribution of pneumonia within the lungs. Impairment of mucociliary clearance and the cough reflex has been associated with increased infection risk and altered radiological patterns, particularly in influenza, smoking-related lung disease, and chronic pulmonary conditions [25,26,27].

More recent evidence has also emphasized the importance of innate and adaptive immune dysfunction in determining disease extent. In particular, impaired alveolar macrophage activity and lymphocyte-mediated responses, especially in immunocompromised patients, have been linked to more extensive and atypical imaging manifestations of pneumonia [28,29,30].

In addition, contemporary studies have shown that established risk factors such as advanced age, chronic lung disease, malnutrition, and patient positioning contribute to heterogeneous radiological patterns ranging from lobar to diffuse or cavitary involvement [31,32].

#### 3.1.4. Host Characteristics

Host-related factors play a major role in determining both susceptibility to pneumonia and its pulmonary distribution patterns. Recent studies have emphasized that age, immune status, chronic respiratory diseases, comorbidities, and body positioning significantly influence the clinical and radiological presentation of pulmonary infections [33].

Age is a major determinant: pediatric and elderly populations exhibit reduced mucociliary clearance and immune efficiency, increasing susceptibility to lobar pneumonia or bronchopneumonia [34,35]. In older adults, dependent lung regions are particularly vulnerable due to reduced ventilation and secretion clearance [36].

Immunosuppression due to chronic diseases, viral infections, organ transplantation, or immunosuppressive therapy increases susceptibility to opportunistic pathogens and may alter typical distribution patterns, including involvement of upper lobe regions like in infections with *Mycobacterium tuberculosis* [37].

Chronic lung diseases such as COPD, asthma, and cystic fibrosis (CF) are associated with structural airway changes and impaired mucus clearance [38]. In CF, thick mucus and basal hypoventilation favor chronic colonization with *Pseudomonas aeruginosa* in the upper lobes, whereas patients with COPD are more susceptible to infections with *Haemophilus influenzae* in the basal regions [39,40].

In addition, systemic comorbidities such as diabetes, malnutrition, and alcohol misuse impair systemic immune responses and tissue repair, increasing the risk of severe or necrotizing pneumonia [41].

Body position further influences the distribution and severity of pneumonia, particularly in aspiration-related disease, where dependent lung regions are most frequently affected [42,43].

#### 3.1.5. Environmental and Exogenous Risk Factors

Exogenous factors significantly contribute to the distribution and severity of pneumonia [44] and include environmental exposure, smoking, inhaled pollutants, infected aerosols, iatrogenic procedures, and conditions related to international travel. Environmental factors can influence host susceptibility by altering airway defense mechanisms and affecting pathogen viability and transmission [45]. Airborne pathogens, dust, and fungal spores in indoor and outdoor environments increase the likelihood of inhalational exposure, particularly in crowded or poorly ventilated settings [46,47,48].

Exposure to cigarette smoke and air pollution impairs mucociliary clearance and promotes airway inflammation, increasing susceptibility to infection, especially in vulnerable populations [49,50].

Healthcare-related procedures such as intubation, bronchoscopy, or mechanical ventilation are associated with nosocomial pneumonia, often affecting the posterior segments of the lower lobes [51]. Altered airflow due to endotracheal valves facilitates colonization of specific areas and increases susceptibility to Gram-negative bacteria and *Staphylococcus aureus* [52].

International travel can expose individuals to uncommon pathogens, leading to infections rarely seen locally and affecting severity and distribution patterns [53].

### 3.2. Imaging Patterns of Pneumonia

This section focuses primarily on bacterial pneumonia, as the most clinically relevant and best-characterized form in relation to imaging patterns, while viral, fungal, and atypical pathogens are included for comparative purposes.

Imaging plays a central role in the evaluation of pneumonia by supporting diagnosis, estimating disease severity, and identifying complications [54,55]. Pulmonary imaging is shifting toward quantitative, AI-driven radiology, with CT, radiomics, and deep learning improving lesion characterization, pathogen differentiation, and diagnostic accuracy in pneumonia [56]. Although radiological appearances are variable and often overlap, several recurring patterns are clinically useful when interpreted together with clinical and microbiological findings [57].

As summarized in Table 1, recognition of these distinct patterns may facilitate early diagnostic orientation, risk stratification, and therapeutic decision-making, but imaging findings alone are usually insufficient for definitive etiological diagnosis.

#### 3.2.1. Lobar Pneumonia

Lobar pneumonia typically involves one pulmonary lobe and is most commonly associated with pathogens that spread rapidly through the alveolar spaces, particularly *Streptococcus pneumoniae*. Imaging usually demonstrates homogeneous, well-demarcated consolidation with air bronchograms [57].

#### 3.2.2. Bronchopneumonia (Lobular Pneumonia)

Bronchopneumonia is characterized by patchy, irregular, multifocal, and often bilateral opacities, frequently with lower lobe predominance [58]. Common pathogens include *Staphylococcus aureus*, *Haemophilus influenzae*, Gram-negative bacilli, and nosocomial pathogens. The peribronchovascular distribution reflects spread from the bronchioles to adjacent alveoli, while the heterogeneous pattern suggests multifocal airway-centered infection, often favored by impaired host defense [59].

#### 3.2.3. Interstitial Pneumonia

Interstitial pneumonia commonly presents with reticular opacities, Kerley lines, interlobular septal thickening, and peribronchovascular infiltrates, and is most often associated with viral infections (e.g., influenza and *SARS-CoV*), atypical pathogens (e.g., *Mycoplasma pneumoniae* and *Chlamydophila pneumoniae*), or hypersensitivity pneumonitis [60]. This pattern reflects predominantly interstitial rather than alveolar inflammation and is often accompanied by dry cough, moderate fever, and limited auscultatory findings [61].

#### 3.2.4. Severe and Complicated Pneumonia

##### Necrotizing Pneumonia

Necrotizing pneumonia represents severe parenchymal destruction caused by highly virulent organisms, including post-influenza *Staphylococcus aureus*, *Klebsiella pneumoniae*, *Pseudomonas aeruginosa*, and anaerobes. Imaging may show hypodense areas with loss of normal alveolar architecture. Clinically, persistent fever, severe systemic toxicity, and poor response to standard antibiotics should raise suspicion [62].

##### Abscessing Pneumonia

Lung abscess is a severe complication characterized by cavitary lesions with air–fluid levels [63], and is commonly associated with aspiration syndromes, anaerobic infections, *Staphylococcus aureus*, or Gram-negative bacilli, often involving dependent lung segments [64].

##### Cavitating Pneumonia

Cavitation may occur in infections caused by *Mycobacterium tuberculosis*, *Staphylococcus aureus*, *Klebsiella pneumoniae*, and fungal pathogens, or in prolonged uncontrolled infections [65]. Cavitary lesions indicate central tissue necrosis and parenchymal destruction and are clinically relevant because they may suggest aggressive disease, chronic infection, or alternative diagnoses [66].

##### Imaging Features of Severe Pneumonia

Extensive consolidation, bulging fissure sign, atelectasis, and hyperinflation are recognized features of severe pneumonia. The bulging fissure sign, classically associated with *Klebsiella pneumoniae*, results from lobar expansion caused by abundant inflammatory exudate, commonly involves the right upper lobe, and is rarely seen if antibiotics are started promptly [67,68]. Atelectasis may occur secondary to bronchial obstruction by mucus, tumors, or a foreign body and can obscure underlying pneumonia [69]. Hyperinflation is more common in children with viral or *Mycoplasma* infections and may indicate associated bronchiolitis [70].

### 3.3. Pathogen-Specific Clinicoradiological Features in Pneumonia

Identifying the causative pathogen is important for understanding lobar predilection and the radiological pattern of pneumonia [12]. Radiological aspects of the disease reflect the interaction between pathogen characteristics, host defense mechanisms, and patient-related factors [71].

#### 3.3.1. *Streptococcus pneumoniae*

*Streptococcus pneumoniae* remains the leading cause of community-acquired pneumonia, and its pulmonary distribution is closely linked to aspiration mechanisms and host anatomical factors [72].

The main route of pulmonary infection is microbial aspiration of colonized oropharyngeal secretions, particularly when local defenses, such as the cough reflex, mucociliary clearance, or epithelial integrity, are compromised [73]. Nasopharyngeal colonization is especially common in children, making the aspiration of colonized secretions a common, sometimes unavoidable, pathway into the lower respiratory tract [74]. In upright or seated individuals, gravity favors deposition in the lung bases, explaining the common involvement of lower lobes [75]. In older adults, right lower lobe predominance is further explained by the shorter, wider, and more vertical right bronchus [13,76].

Patients at increased risk are children, because of intense nasopharyngeal colonization; older adults with impaired mucociliary clearance and reduced cough reflex; and individuals with diabetes mellitus, in whom dysfunction of innate and adaptive immunity enhances bacterial invasion [77,78].

**Figure 1:**
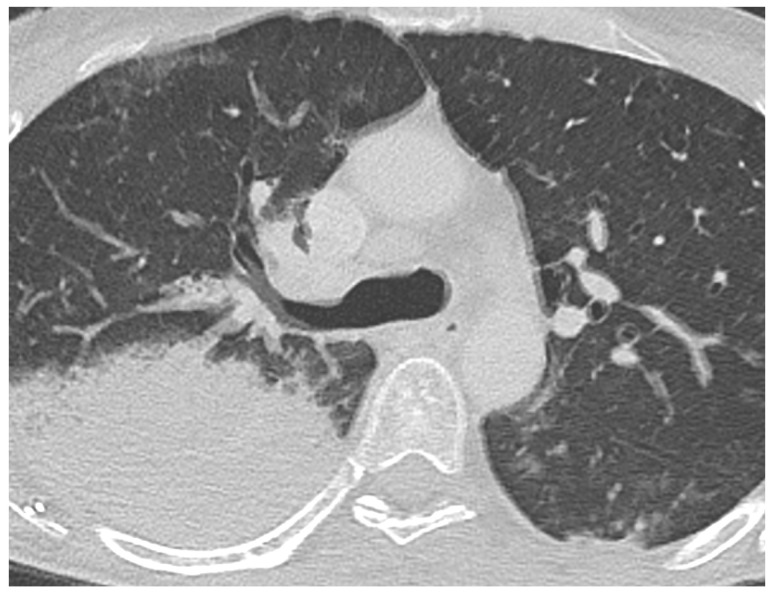
Chest CT in an elderly patient with *Streptococcus pneumoniae* infection: unilateral homogeneous consolidation in the right lower lobe, consistent with lobar pneumonia. Representative image from the anonymized institutional radiology archive of University Hospital Frankfurt.

Imaging typically demonstrates rapidly developing homogeneous lobar consolidation, which may be unilateral or bilateral (Figure 1).

Clinically, pneumococcal pneumonia often follows a viral respiratory infection and presents abruptly with chills, high fever, productive cough, and pleuritic chest pain. Pleural effusion is common, whereas abscess formation, pericarditis, and bacteremia are less frequent. Outcome depends on comorbidities, the extent of infection, complications, and the prompt initiation of effective antibiotic therapy [79].

#### 3.3.2. *Klebsiella pneumoniae*

*Klebsiella pneumoniae* is a Gram-negative bacillus classically associated with severe pneumonia involving the upper lobes, especially the right upper lobe, and is more frequent in patients with alcoholism, malnutrition, neurological disease, or prolonged immobilization [80].

This distribution reflects impaired airway protection and recurrent aspiration. Chronic alcoholism reduces the cough reflex, alters nutritional status, and weakens immune defenses, thereby increasing aspiration risk [81,82]. Prolonged immobilization, neurological diseases, sedation, or coma further promote colonization by repeated microaspirations [83]. The anatomy of the right main bronchus also facilitates preferential entry into the right upper lung [84].

A prominent feature of *Klebsiella* infection is the production of copious mucoid exudate rich in capsular polysaccharides, resulting in dense lobar consolidation and rapid progression [85,86]. Necrosis, abscess formation, and bacteremia are common complications [87].

Clinically, patients often present with high fever, marked systemic toxicity, productive cough with thick mucoid or “currant jelly” sputum, pleuritic chest pain, and respiratory failure in severe cases [88].

**Figure 2:**
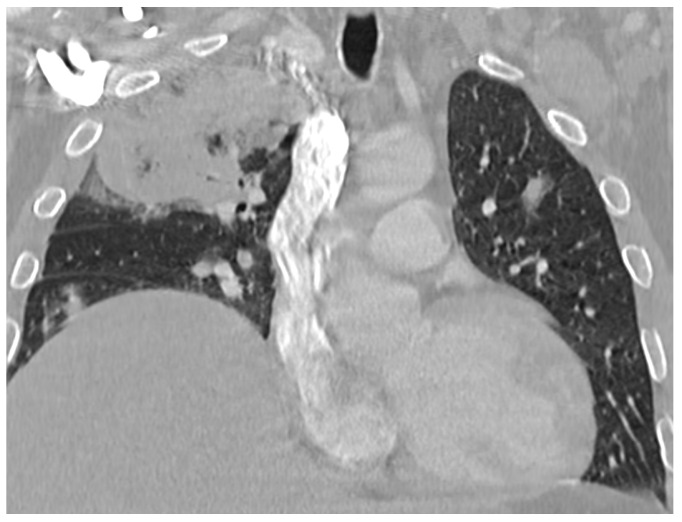
Chest CT opacity in a patient with prolonged immobilization and *Klebsiella pneumonia*: consolidation in the right upper lobe with slight bulging of the medial segment of the right horizontal fissure and surrounding ground-glass. Representative image from the anonymized institutional radiology archive of University Hospital Frankfurt.

Chest CT may show expansive consolidation with the classic bulging fissure sign, caused by lobar enlargement and fissural displacement from abundant exudate (Figure 2).

#### 3.3.3. *Staphylococcus aureus*

*Staphylococcus aureus* is among the most aggressive etiological agents of pneumonia, particularly after influenza infection [89]. Viral damage to the respiratory epithelium disrupts cilia, impairs mucociliary clearance, and weakens mucosal barriers, thereby facilitating secondary bacterial invasion, especially in the upper bronchial segments where viral replication is most intense [90]. Upper lobe predominance may also relate to preserved ventilation and higher regional oxygen tension, conditions favorable for the growth of aerobic organisms such as *Staphylococcus aureus* [91,92]. After colonization, the pathogen can invade deeply and provoke an intense inflammatory response [93].

Clinically, staphylococcal pneumonia usually presents abruptly with high fever, chills, severe alteration of general condition, progressive dyspnea, and rapidly productive cough. Hemoptysis and early respiratory failure may also occur [93].

**Figure 3:**
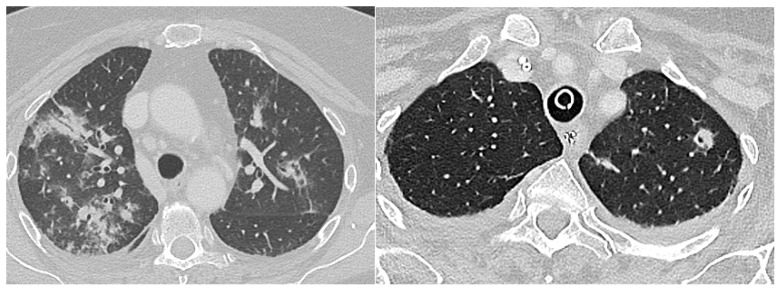
Chest CT. (**Left**): small patchy consolidations in the upper lobes, more pronounced on the right side, with mild associated ground-glass opacity and air bronchograms, suggestive of Staphylococcus aureus pneumonia. (**Right**): small consolidation with a tiny cavity in the left upper lobe in another patient with *Staphylococcus aureus* infection. Representative image from the anonymized institutional radiology archive of University Hospital Frankfurt.

Imaging frequently displays a necrotizing pattern, caused by toxin-mediated tissue injury, particularly with Panton–Valentine leukocidin (PVL)-producing strains, which induces leukocyte destruction and extensive parenchymal necrosis [94]. Radiological findings include multiple cavities (Figure 3), areas of liquefaction, small or large lung abscesses, and the characteristic pneumatocele—transient air-filled spaces resulting from alveolar wall destruction. Empyema, pneumothorax, and acute respiratory distress syndrome are important complications [95]. Overall, the severe clinicoradiological presentation results from the combined effects of epithelial injury, favorable regional physiology, and intrinsic bacterial virulence [96].

#### 3.3.4. *Pseudomonas aeruginosa*

*Pseudomonas aeruginosa* is a major pathogen in pulmonary infections among patients with cystic fibrosis (CF), often showing upper lobe predominance [97,98].

In CF, CFTR (cystic fibrosis transmembrane conductance regulator) dysfunction leads to the production of extremely viscous, sticky mucus that is difficult to clear. Although all lung regions are affected, altered respiratory mechanics and progressive bronchial remodeling may reduce effective ventilation in dependent regions, while the upper lobes remain relatively aerated but prone to mucus retention and dehydration. These conditions favor bacterial adhesion and persistence [99,100].

Chronic colonization promotes biofilm production, which protects bacteria from antibiotics and host immunity [101]. Persistent neutrophilic inflammation further damages airways and perpetuates a cycle of mucus retention, infection, and structural injury [102,103].

Clinically, *Pseudomonas aeruginosa* pneumonia can develop insidiously or abruptly, presenting with high fever, chills, productive cough with purulent or greenish sputum, dyspnea, pleuritic chest pain, and occasionally hemoptysis, systemic toxicity, hypotension, or septic shock [98].

**Figure 4:**
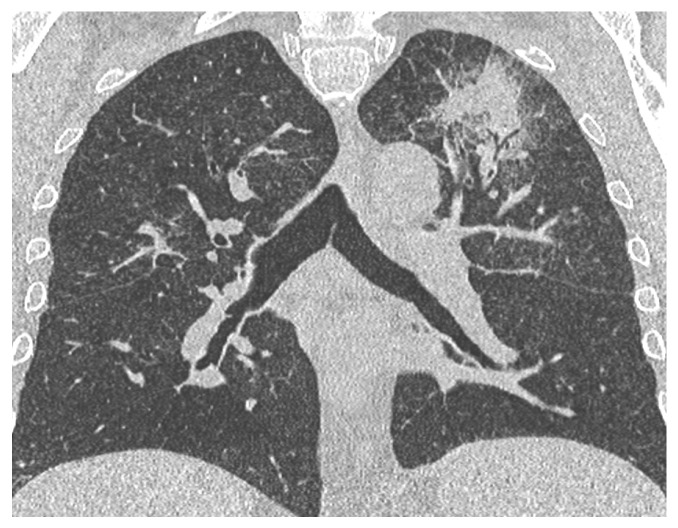
Chest CT: consolidation in the left upper lobe with bronchiectasis and positive air bronchograms, consistent with *Pseudomonas aeruginosa* pneumonia. Representative image from the anonymized institutional radiology archive of University Hospital Frankfurt.

Chest CT typically demonstrates diffuse bronchopneumonia with upper lobe bronchiectasis, persistent opacities, and parenchymal destruction [104] (Figure 4).

#### 3.3.5. *Haemophilus influenzae*

*Haemophilus influenzae* is a frequent pathogen in acute respiratory infections and exacerbations of chronic lung diseases [105], and its biological characteristics and behavior in the respiratory tract help explain its predilection for the lower lobes [106].

The organism primarily affects small and medium bronchi, leading to bronchiolitis and bronchitis with mucus hypersecretion, edema, and secretion stasis. Because mucociliary clearance is less efficient in dependent lung regions, infected secretions accumulate preferentially in the lower lobes [107,108].

This mechanism is particularly relevant in COPD, where bronchial remodeling, inflammation, and emphysematous damage further impair mucociliary clearance and promote chronic colonization by *H. influenzae*. Recurrent lower lobe infections are therefore common [109,110]. Clinical presentation is often more subacute, with fever, chills, productive cough with purulent sputum, dyspnea, and pleuritic chest pain, accompanied by malaise, fatigue, and decreased exercise tolerance [106].

**Figure 5:**
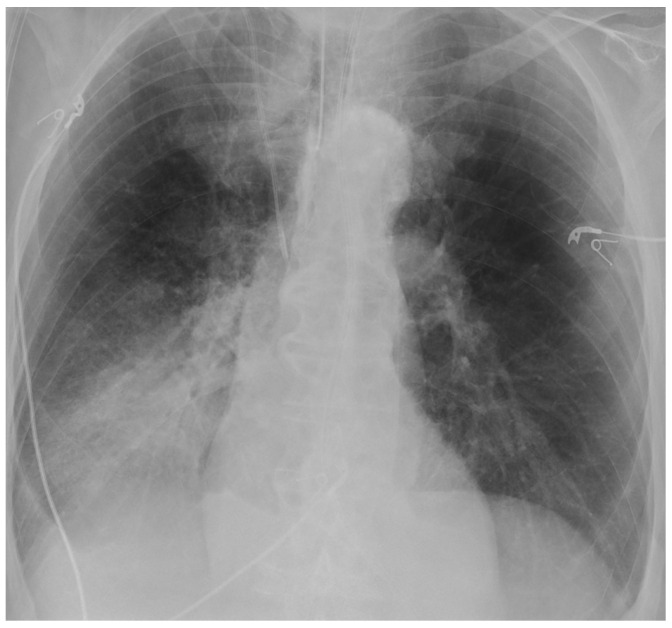
Chest radiograph in an elderly patient with COPD with *Haemophilus influenzae* pneumonia: homogeneous consolidation in the right lower lobe with air bronchograms. Representative image from the anonymized institutional radiology archive of University Hospital Frankfurt.

Chest CT findings include bronchial wall thickening, centrilobular nodules, and ground-glass opacities, similar to those seen in *Mycoplasma pneumoniae* infections, which can make differentiation challenging. Lobar consolidations may develop in advanced disease or in older patients, and infection frequently precipitates COPD exacerbations (Figure 5). Mortality is generally moderate, although complications require prompt management [111].

In bacterial pneumonia, although overlap exists, some recurring clinicoradiological associations are clinically useful: lower lobe homogeneous consolidation suggests *S. pneumoniae* or *H. influenzae*, upper lobe expansive necrotic consolidation indicates *Klebsiella pneumoniae*, multifocal cavitating disease suggests *S. aureus*, and chronic upper lobe bronchiectatic changes favor *P. aeruginosa* in cystic fibrosis. Imaging findings should always be interpreted in conjunction with host factors and microbiological data.

#### 3.3.6. Atypical Pneumonias

Atypical pneumonias caused by *Mycoplasma pneumoniae*, *Chlamydophila pneumoniae*, *Legionella pneumophila*, and *Coxiella burnetii* are characterized by predominantly interstitial, diffuse, and often bilateral imaging patterns rather than classic lobar consolidation [112]. In contrast to typical pyogenic bacteria, these organisms commonly involve the respiratory epithelium, bronchioles, or interstitium, resulting in less exudative alveolar filling and a weaker correlation with specific lobar anatomy [60,113,114].

**Figure 6:**
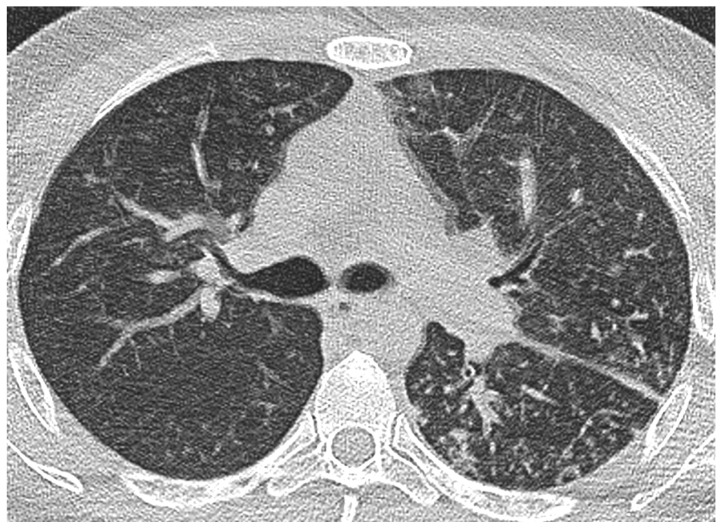
Chest CT: unilateral interstitial infiltrates, appearing as diffuse reticular basal bronchopneumonic opacities, consistent with atypical pneumonia due to *Mycoplasma pneumoniae*. Representative image from the anonymized institutional radiology archive of University Hospital Frankfurt.

*Mycoplasma pneumoniae* has marked tropism for bronchial and bronchiolar epithelium, producing airway-centered and interstitial inflammation rather than dense alveolar consolidation. Clinical presentation is typically mild to moderate, with dry cough, fever, headache, and fatigue. Extrapulmonary manifestations may include cutaneous eruptions, hemolytic anemia, neurological involvement (e.g., meningitis and encephalopathy), cardiac manifestations (e.g., myocarditis), and arthralgias [115,116]. Chest CT commonly shows diffuse reticular or perihilar and basal bronchopneumonic opacities. Unilateral disease is common, while bilateral involvement occurs less frequently (Figure 6) [117].

Similarly, *Chlamydophila pneumoniae* causes slowly progressive respiratory infection with persistent dry cough, moderate fever, hoarseness, headache, and fatigue. Extrapulmonary features may include pharyngitis, sinusitis, otitis, skin rashes, arthralgias, and rarely, neurological or cardiac involvement [112].

**Figure 7:**
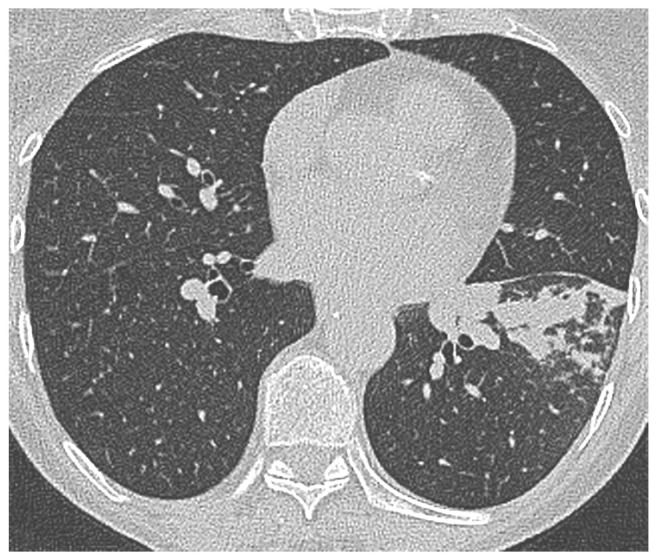
Chest CT: unilateral interstitial infiltrates with mild segmental patchy bronchopneumonic opacities and ground-glass changes, suggestive of atypical pneumonia compatible with *Chlamydophila pneumoniae*. Representative image from the anonymized institutional radiology archive of University Hospital Frankfurt.

Because multiple pulmonary segments may be involved simultaneously, imaging often reveals bilateral patchy infiltrates rather than a single focal lobar opacity. Typical chest CT findings include small subsegmental consolidations, ground-glass opacities, and limited lobar involvement. Pleural effusions are uncommon but may occur (Figure 7) [118].

**Figure 8:**
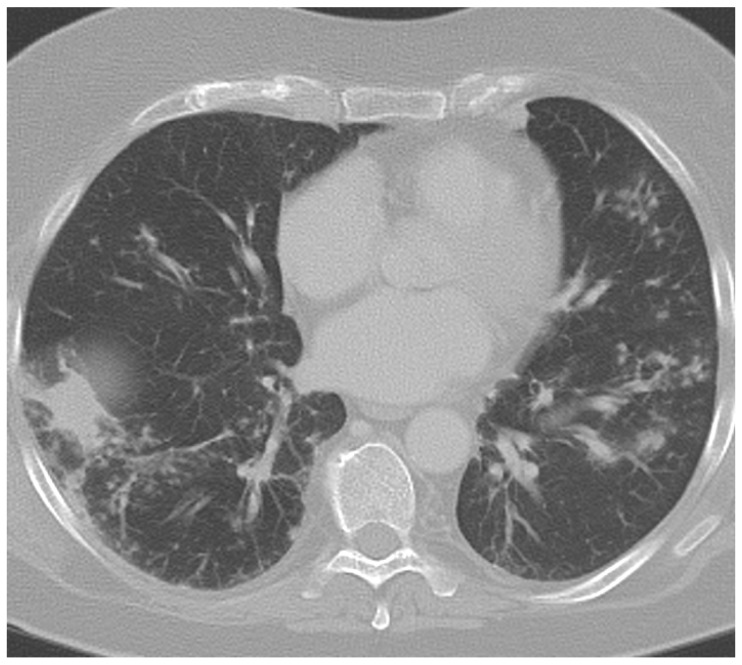
Chest CT in an elderly smoker with cardiovascular disease and atypical pneumonia compatible with *Legionella pneumophila*: segmental consolidation in the right lower lobe with surrounding ground-glass opacities, associated with small subpleural nodules diffusely distributed bilaterally.

Pneumonia caused by *Legionella pneumophila* is frequently associated with recent travel (e.g., hotels, cruise ships, and healthcare facilities) or occupational exposure among plumbers and maintenance workers. Symptoms typically develop 2–10 days after exposure and include fever, chills, myalgias, dry cough, dyspnea, chest pain, gastrointestinal complaints, and neurological manifestations [119]. Although clinically severe, early imaging may initially appear disproportionally subtle. Chest CT commonly demonstrates multifocal or bilateral consolidations with surrounding ground-glass opacities, usually in the middle and lower lung zones (Figure 8). Pleural effusions are relatively common, and radiologic resolution is often delayed [120].

Smokers, older adults, patients with cardiovascular disease, and immunocompromised hosts are more likely to develop extensive and bilateral involvement [121,122].

*Coxiella burnetii* is an obligate intracellular pathogen with tropism for alveolar macrophages and endothelial cells, producing predominantly interstitial pulmonary inflammation [123]. Patients often present with high-grade fever, dry cough, dyspnea, severe headache, and pronounced systemic symptoms [124]. Extrapulmonary manifestations are common and include hepatitis, gastrointestinal symptoms, neurological involvement (meningoencephalitis), cardiac complications such as myocarditis or endocarditis, and hematological abnormalities [125]. Chest CT findings usually include bilateral or diffuse reticulonodular opacities, peribronchovascular thickening, and patchy ground-glass opacities. Focal consolidations may occur, whereas pleural effusions and lymphadenopathy are uncommon [126].

#### 3.3.7. Opportunistic and Granulomatous Pneumonias

Opportunistic and granulomatous pneumonias occur predominantly in immunocompromised patients or those with chronic lung disease, and major pathogens include *Aspergillus*, *Histoplasma*, *Pneumocystis jirovecii*, and *Mycobacterium tuberculosis*. Imaging, especially CT, is essential for detecting characteristic patterns, assessing disease extent, and guiding treatment decisions.

**Figure 9:**
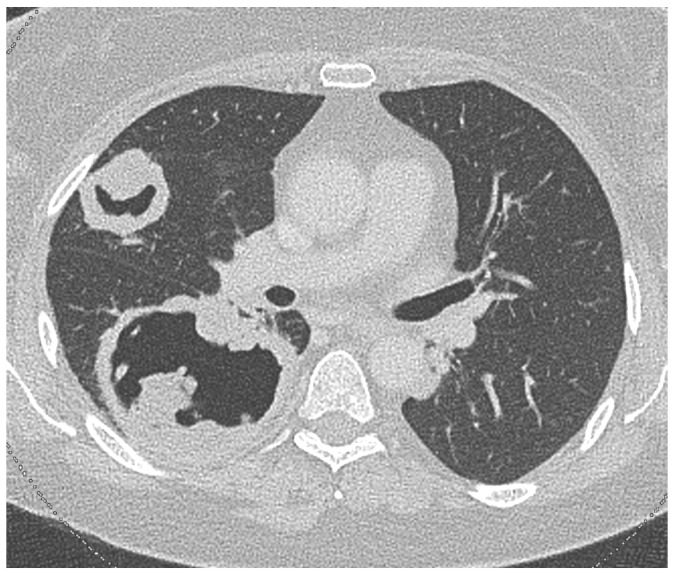
Chest CT: two cavitary lesions in the right upper and lower lobes, with the lower cavity containing a fungal mass with a thickened wall, consistent with aspergilloma. Findings are suggestive of pulmonary aspergillosis. Representative image from the anonymized institutional radiology archive of University Hospital Frankfurt.

*Aspergillus* infections mainly affect immunocompromised patients with prolonged neutropenia, solid organ transplantation, or chronic lung diseases [127]. Invasive disease may cause vascular invasion, infarction, hemoptysis, and necrotizing pneumonia [128]. Chest CT typically demonstrates nodules with surrounding ground-glass hemorrhage (halo sign), followed later by cavitation and the air-crescent sign during recovery. Chronic pulmonary aspergillosis usually manifests as upper lobe cavities containing a mobile intracavitary mass (aspergilloma) with pleural thickening (Figure 9) [129,130].

**Figure 10:**
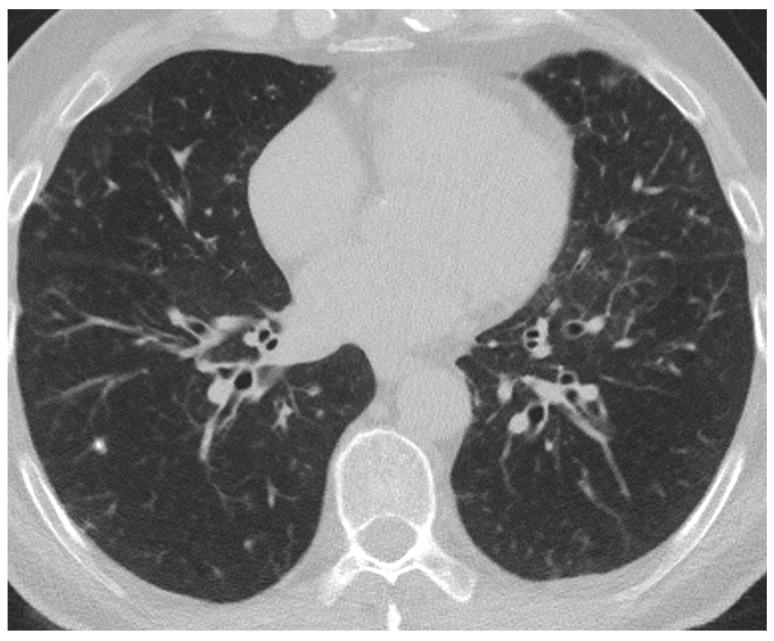
Chest CT: diffuse nodular infiltrates in the right lung, with areas of calcification in the superior segment of the right lower lobe, consistent with pulmonary histoplasmosis. Representative image from the anonymized institutional radiology archive of University Hospital Frankfurt.

*Histoplasma capsulatum* infection follows inhalation of spores from soil contaminated by bird or bat droppings [131], and disease severity depends on the inoculum size and host immunity. Many infections are asymptomatic or mild, whereas immunocompromised patients may develop disseminated disease [132]. Chest CT findings include diffuse interstitial or nodular infiltrates, accompanied by calcifications in chronic stages (Figure 10). No consistent lobar predilection is observed [133].

*Pneumocystis jirovecii* (formerly *Pneumocystis carinii*) pneumonia occurs primarily in patients with HIV/AIDS, organ transplants, or hematologic malignancy, or those undergoing immunosuppressive therapy [134]. Symptoms often progress subacutely with dyspnea, dry cough, and fever [135].

**Figure 11:**
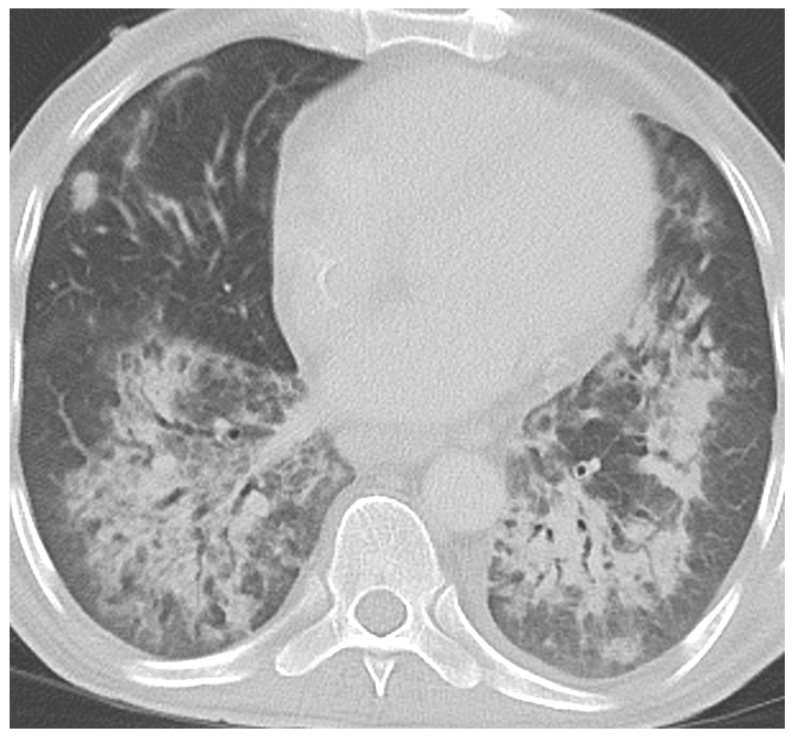
Chest CT: bilateral perihilar infiltrates and ground-glass opacities with subpleural sparing, without specific lobar predilection, in a patient with AIDS, consistent with *Pneumocystis jirovecii* pneumonia (PJP). Representative image from the anonymized institutional radiology archive of University Hospital Frankfurt.

Chest CT classically shows diffuse bilateral ground-glass opacities with perihilar predominance and relative subpleural sparing. Thin-walled cysts may develop in advanced disease and predispose to pneumothorax (Figure 11) [136].

*Mycobacterium tuberculosis* demonstrates a characteristic upper lobe predilection related to its aerobic metabolism and favorable growth in highly ventilated, oxygen-rich apical lung regions [137,138,139,140]. Reduced regional perfusion and an altered immune microenvironment may further facilitate bacillary persistence [141].

Primary tuberculosis can affect immunocompetent individuals, whereas reactivation and progressive forms are more common in immunocompromised patients with HIV, diabetes, or malnutrition, or those undergoing immunosuppressive therapy [142]. Clinical presentation includes chronic cough, fever, night sweats, weight loss, hemoptysis, and fatigue [143].

**Figure 12:**
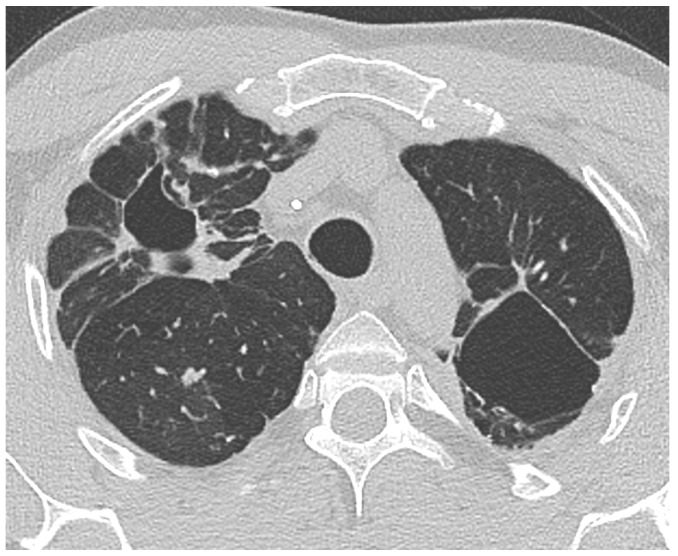
Chest CT: multiple thin-walled cavitary lesions in both upper lobes, consistent with active pulmonary tuberculosis. Representative image from the anonymized institutional radiology archive of University Hospital Frankfurt.

Chest CT findings are heterogeneous but classically include apical cavitary lesions, centrilobular nodules, tree-in-bud opacities, consolidation, and fibronodular scarring. Lower lobe disease, lymphadenopathy, or pleural effusion is more common in children and immunocompromised adults (Figure 12) [144].

The typical upper lobe cavitary pattern remains a valuable imaging clue for differentiating tuberculosis from most bacterial pneumonias, which more often involve dependent or lower lung regions [145].

## 4. Discussion

This narrative review summarizes current evidence on the clinical and radiological determinants of pneumonia, emphasizing the complex interaction between pathogens, host immune status, and anatomical factors. Although the route of infection may help predict likely pathogens and radiological patterns, disease distribution remains influenced by lung anatomy, physiological gradients, and patient positioning in a probabilistic rather than deterministic manner. Host immune competence is a major determinant of susceptibility and disease severity; however, its interaction with microbial virulence and structural lung abnormalities remains multifactorial. Consequently, imaging findings alone are insufficient for etiological diagnosis without integration of clinical and microbiological data [146].

Imaging plays a central role in diagnosis, severity assessment, and therapeutic monitoring, although radiological findings are frequently non-specific. Lobar pneumonia commonly presents as homogeneous consolidation with air bronchograms, whereas bronchopneumonia typically demonstrates patchy peribronchovascular opacities. Interstitial pneumonia is more often associated with diffuse reticular or ground-glass abnormalities. In severe cases, infection may progress to necrosis, abscess formation, or cavitation. These patterns may support diagnostic orientation but should be interpreted in conjunction with clinical and microbiological findings [147].

Although no imaging pattern in bacterial pneumonia is pathogen-specific, recurring radiological associations may still support early diagnostic hypotheses. In this context, Table 2 summarizes characteristic associations between etiological agents, predominant CT patterns, preferential lobar or segmental distribution, and key imaging findings relevant to diagnosis of bacterial pneumonias.

Atypical pathogens, including *Mycoplasma pneumoniae*, *Chlamydophila pneumoniae*, *Legionella pneumophila*, and *Coxiella burnetii*, are more frequently associated with diffuse interstitial or mixed alveolar–interstitial patterns. Their principal CT manifestations and the most relevant differentiating imaging features are summarized in Table 3.

Opportunistic infections may demonstrate characteristic but non-specific findings, such as halo signs in aspergillosis, diffuse ground-glass opacities in *Pneumocystis jirovecii* pneumonia, granulomatous nodules in histoplasmosis, or apical cavitary lesions in tuberculosis. Table 4 summarizes the principal CT findings of these infections.

However, these findings should always be interpreted within the clinical and epidemiological context. Integration of clinical, microbiological, and radiological data improves diagnostic confidence, guides therapy and supports better outcomes [148].

Integration of radiological distribution, clinical history, and patient risk factors may improve early diagnostic confidence prior to microbiological confirmation. Lower-lobe consolidation in otherwise healthy adults is frequently associated with *Streptococcus pneumoniae*, whereas upper-lobe disease in patients with cystic fibrosis may raise suspicion for *Pseudomonas aeruginosa*. Similarly, aspiration pneumonia predominantly affects dependent lung regions, while multifocal consolidation and cavitation may suggest necrotizing infection caused by *Staphylococcus aureus* or Gram-negative bacilli. Nevertheless, these associations are not pathogen-specific.

The COVID-19 pandemic further emphasized the considerable overlap between viral and atypical imaging manifestations, particularly bilateral ground-glass opacities and multifocal involvement [149].

Integrated clinical and radiological assessment may facilitate early empirical treatment decisions; however, patient outcomes remain strongly influenced by disease severity, comorbidities, immune status, and healthcare setting [150]. Microbiological confirmation remains essential in non-responding patients or in cases demonstrating radiological progression, including cavitation, necrosis, or atypical distribution. Such findings should prompt reassessment of resistant pathogens, opportunistic infections, fungal disease, mycobacterial infection, or alternative non-infectious diagnoses, including malignancy. Although certain radiological signs, such as the bulging fissure sign in *Klebsiella pneumoniae* infection, may be suggestive, they lack sufficient specificity for definitive diagnosis [150,151].

In selected cases, persistent or progressive abnormalities despite appropriate therapy should raise suspicion for non-infectious etiologies. Ground-glass nodules may represent inflammatory lesions or early neoplastic disease [152]; therefore, CT follow-up and histopathological evaluation remain important when lesion progression or development of solid components is observed [153]. 

**Figure 13:**
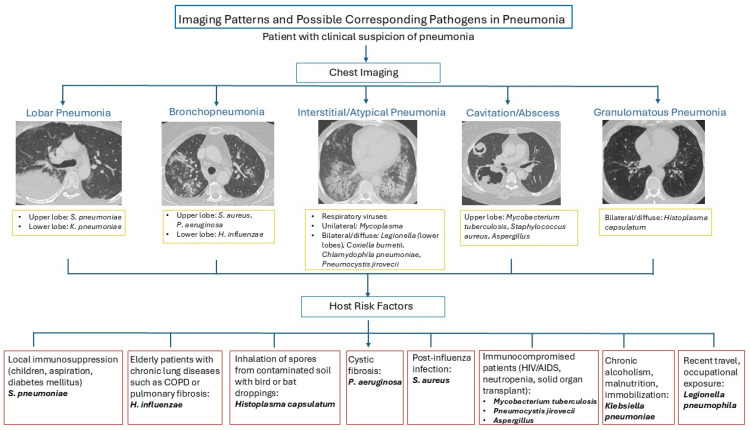
Typical CT patterns in relation to potential pathogens and relevant patient risk profiles.

Based on the integrated analysis of the reviewed literature, Figure 13 proposes a practical clinicoradiological framework correlating typical CT patterns with potential pathogens, host immune status, and relevant patient risk factors.

Overall, imaging findings in pneumonia are rarely pathognomonic. Accurate interpretation requires integration of radiological, clinical, epidemiological, and laboratory data, particularly in acute-care settings where rapid therapeutic decisions are necessary.

Preventive strategies remain essential for reducing pneumonia burden. Vaccination against influenza and pneumococcal disease has been shown to reduce hospitalization rates, severe infections, and mortality in high-risk populations [154]. Early identification of vulnerable patients enables timely preventive interventions and may reduce disease severity and complications.

Recent advances in thoracic CT, molecular diagnostics, and the increased recognition of opportunistic and viral pneumonias during the COVID-19 pandemic have further emphasized the importance of integrated clinicoradiological assessment and early risk stratification in pneumonia management.

Overall, effective pneumonia management requires the integration of clinical, microbiological, and imaging data to support individualized, evidence-based decision-making [155].

### 4.1. Limitations

This review has several limitations that should be considered when interpreting the findings.

First, the article is that of a narrative based on a structured literature search rather than a systematic review or meta-analysis. Although a comprehensive search strategy was applied, no formal PRISMA-based protocol was used; therefore, selection bias cannot be excluded, and the reproducibility of the search strategy may be limited.

Second, the included literature is largely based on European epidemiological data, which may limit generalizability to other regions with different pathogen distributions, healthcare systems, vaccination coverage, and antimicrobial resistance patterns.

Third, heterogeneity across the included studies—regarding differences in populations, imaging modalities, and diagnostic criteria—limits comparability and may affect the consistency of reported associations between imaging patterns. This heterogeneity was assessed qualitatively rather than quantified statistically, as no meta-analysis was performed.

### 4.2. Future Perspectives

Future research should aim to improve diagnostic accuracy through multimodal and data-driven approaches.

Artificial intelligence-assisted image analysis represents a promising tool to enhance radiological interpretation in pneumonia. In particular, deep learning-based systems may support its differential diagnosis by improving the detection and classification of characteristic CT patterns. Emerging evidence suggests that such computer-aided diagnostic (CAD) approaches can reduce interobserver variability and improve diagnostic consistency, especially in complex cases and immunocompromised patients [156]. In addition, AI-assisted tools may contribute to the differentiation between bacterial, viral, and atypical infectious patterns, thereby supporting earlier and more structured clinical decision-making in acute care settings.

Combining imaging findings with laboratory biomarkers and clinical scoring systems may further enhance etiological differentiation and support more targeted therapeutic strategies. The integration of multimodal datasets could improve risk stratification and enable earlier identification of patients at risk for severe disease progression.

The development of validated predictive models integrating clinical, radiological, and microbiological data should be prioritized. These models require evaluation in prospective multicenter studies to determine their impact on diagnostic performance, treatment selection, and patient outcomes.

Overall, future progress in pneumonia management will depend on a multidisciplinary approach integrating radiology, clinical medicine, microbiology, and computational methods.

## 5. Conclusions

This review highlights that the distribution of pulmonary infections is not random, but reflects complex interactions between pathogen characteristics, host factors, anatomical structures, and local defense mechanisms. Understanding these relationships may facilitate a more integrated clinicoradiological interpretation of pneumonia and improve diagnostic assessment.

In current clinical practice, recognition of characteristic topographic and radiological patterns may assist in narrowing differential diagnostic hypotheses, guiding empirical therapy, identifying high-risk patients, and monitoring disease progression or complications. However, imaging findings remain non-specific and should always be interpreted in conjunction with clinical, laboratory, and microbiological findings.

Although the heterogeneity of the available literature limits direct etiological generalization, integrating clinical, microbiological, and imaging information may support more personalized pneumonia management, facilitate earlier therapeutic decision-making, and ultimately improve patient outcomes.

## Figures and Tables

**Table 1:** Clinicoradiological patterns of pneumonia: imaging features, etiology, and clinical presentation [57].

Pneumonia Pattern	Key Imaging Features	Common Etiologic Agents	Typical Clinical Characteristics
Lobar pneumonia	Homogeneous, well-defined lobar consolidation with air bronchograms; occasional bulging fissure in severe cases	*Streptococcus pneumoniae*, *Klebsiella pneumoniae*	Acute onset, chills, high fever, productive cough, pleuritic chest pain
Bronchopneumonia	Patchy multifocal, ill-defined opacities with peribronchovascular distribution, often bilateral and basal	*Staphylococcus aureus*, *Haemophilus influenzae*, Gram-negative bacilli	Subacute onset, moderate fever, productive cough, dyspnea; common in elderly/comorbid patients
Interstitial pneumonia	Reticular opacities, interlobular septal thickening, peribronchovascular infiltrates, ground-glass areas	Respiratory viruses, *Mycoplasma pneumoniae*, *Legionella pneumophila*, *Chlamydophila pneumoniae*, *Coxiella burnetii*	Dry cough, low-to-moderate fever, dyspnea, extrapulmonary symptoms; limited auscultatory findings
Necrotizing pneumonia	Hypodense parenchymal areas, loss of normal alveolar architecture, early cavitation	Post-influenza *Staphylococcus aureus*, *Klebsiella pneumoniae*, *Pseudomonas aeruginosa*, anaerobes	Severe systemic toxicity, persistent fever, rapid clinical deterioration, poor response to standard therapy
Abscessing pneumonia	Cavitary lesions with air–fluid level, typically in dependent lung segments	Anaerobes, aspiration-related pathogens, *Staphylococcus aureus*, Gram-negative bacilli	Prolonged fever, foul sputum, weight loss
Cavitating pneumonia	Thick-walled cavities indicating central necrosis and parenchymal destruction	*Mycobacterium tuberculosis*, *Staphylococcus aureus*, *Klebsiella pneumoniae*, fungi	Chronic cough, hemoptysis, fever, night sweats, progressive clinical course
Severe pneumonia	Extensive consolidation, bulging fissure sign, atelectasis, hyperinflation (children)	*Klebsiella pneumoniae*, severe bacterial or viral pathogens	Acute respiratory failure, hypoxemia, high risk of complications, intensive care unit admission

**Table 2:** Pathogen-specific lobar distribution and characteristic CT features in bacterial pneumonia.

Etiologic Agent	Lobar/Segmental Distribution	Key CT Features	Predominant CT Pattern
*Streptococcus pneumoniae*	Lower lobes, posterior basal segments (right > left)	Homogeneous air-space consolidation with air bronchograms; sharply marginated lobar involvement; frequent parapneumonic pleural effusion	Lobar consolidation
*Klebsiella pneumoniae*	Upper lobes, especially the right upper lobe	High-attenuation consolidation; lobar expansion with bulging fissure sign; internal low-attenuation areas indicating necrosis; early cavitation	Lobar consolidation
*Staphylococcus aureus*	Upper lobes, multifocal	Patchy consolidations with cavitation, pneumatoceles, air–fluid levels; rapid progression; associated empyema or pneumothorax	Necrotizing bronchopneumonia
*Pseudomonas aeruginosa*	Upper lobes in cystic fibrosis or diffuse in non-CF individuals	Centrilobular nodules, patchy consolidations, bronchiectasis, tree-in-bud opacities; parenchymal destruction in advanced disease	Bronchopneumonia
*Haemophilus influenzae*	Lower lobes	Bronchial wall thickening, centrolobular nodules, ground-glass opacities; segmental consolidation in advanced cases	Bronchocentric pneumonia

**Table 3:** CT features of atypical pneumonias (interstitial-predominant patterns).

Etiologic Agent	Dominant CT Pattern	Distribution	Characteristic CT Findings
*Mycoplasma pneumoniae*	Interstitial pneumonia ± bronchopneumonia	Perihilar and basal, often unilateral	Ground-glass opacities, reticular pattern, peribronchovascular thickening; centrilobular nodules; mild lymphoadenopathy
*Chlamydophila pneumoniae*	Patchy interstitial pneumoniae	Multisegmental, frequently bilateral	Small subsegmental consolidations; ground-glass attenuation; limited lobar involvement; occasional pleural effusion
*Legionella pneumophila*	Multifocal pneumoniae with an interstitial component	Bilateral, middle and lower lung zones	Rapid progressive consolidations; surrounding ground-glass opacities; early pleural effusions; delayed radiologic resolution
*Coxiella burnetii*	Interstitial pneumoniae	Diffuse or bilateral	Reticulonodular opacities; peribronchovascular thickening; patchy ground-glass opacities; focal consolidations are uncommon; lymphoadenopathy is rare

**Table 4:** CT imaging features of opportunistic, fungal, and granulomatous pneumonias.

Etiologic Agent	Dominant CT Pattern	Lobar Predilection	Defining CT Signs
*Aspergillus* spp. (invasive)	Angioinvasive pneumonia	Upper lobes	Nodules with surrounding ground-glass hemorrhage (halo sign); cavitation with air-crescent during recovery
Chronic pulmonary aspergillosis	Cavitary disease	Upper lobes	Thick-walled cavities; intracavitary soft-tissue mass (aspergilloma); adjacent pleural thickening
*Histoplasma capsulatum*	Granulomatous pneumonia	No consistent predilection	Diffuse nodular or reticulonodular pattern; calcified granulomas or lymph nodes in chronic phase
*Pneumocystis jirovecii*	Diffuse interstitial pneumonia	Perihilar, bilateral	Symmetric, ground-glass opacities; subpleural sparing; cystic changes in advanced disease
*Mycobacterium tuberculosis*	Cavitary granulomatous pneumonia	Apical and posterior upper lobes	Thick-walled cavities; centrilobular nodules with tree-in-bud pattern; fibronodular opacities

## Data Availability

The original contributions presented in this study are included in the article. Further inquiries can be directed to the corresponding author.

## Abbreviations

The following abbreviations are used in this manuscript:

AIDS	Acquired immune deficiency syndrome
CAP	Community-acquired pneumonia
CF	Cystic fibrosis
CFTR	Cystic fibrosis transmembrane conductance regulator
CHF	Congestive heart failure
CO_2_	Carbon dioxide
COPD	Chronic obstructive pulmonary disease
CT	Computed tomography
EU	European Union
GGNs	Ground-glass nodules
HIV	Human immunodeficiency virus
MeSH	Medical subject headings
PCR	Polymerase chain reaction
PJP	*Pneumocystis jirovecii* pneumonia
PVL	Panton–valentine leukocidin
SARS-CoV	Severe acute respiratory syndrome coronavirus
US	United States
VAP	Ventilator-associated pneumonia
